# Genetic evaluation of longevity in Australian Angus cattle using random regression models

**DOI:** 10.1093/jas/skaf035

**Published:** 2025-02-08

**Authors:** Hassan Aliloo, Julius H J van der Werf, Samuel A Clark

**Affiliations:** School of Environmental and Rural Science, University of New England, Armidale, NSW, Australia; School of Environmental and Rural Science, University of New England, Armidale, NSW, Australia; School of Environmental and Rural Science, University of New England, Armidale, NSW, Australia

**Keywords:** beef cattle, genetic selection, longevity, random regression, stayability, survival

## Abstract

Cow longevity is an economically important trait for beef breeders directly impacting the profitability and sustainability of beef cattle production systems. Despite its great importance, early selection for longevity is complex because the true longevity of a cow is not known until the end of her life. In this study, we aimed to estimate variance components and genetic parameters for 2 binary measures of cow longevity in Australian Angus cattle population. Traditional longevity (TL) represented the cow’s ability to avoid culling after the first calving while functional longevity (FL) also accounted for calving events while the cow was present in the herd. Five datasets consisting of animals culled for different reasons were created and evaluated separately to compare the estimates of variance components and genetic parameters. We also investigated the impact of censored data on estimated breeding values (EBV) of bulls with different proportions of active daughters. A single-trait random regression model using a Bayesian Gibbs sampler was applied to both longevity traits and all 5 culling reason groups between ages 2 to 11 yr. The heritabilites were generally low and ranged between 0.02 to 0.19 for TL and between 0.02 to 0.20 for FL traits. The peak of heritabilites were found between ages 4 to 6 yr for both longevity measures. The low estimates of genetic correlations between ages at the beginning and end of the trajectory in all culling reason groups indicated that longevity evaluated at early and late stages of life are not genetically the same traits. The EBV of sires with active daughters were underestimated when the censored data was excluded from the analysis. The negative impact of censoring was larger for younger sires who had a larger proportion of active daughters. Our results indicate the additive genetic component has a sizeable contribution to the variability of longevity in Australian Angus cattle and therefore, the genetic improvement of longevity can be achieved if longevity is considered as a long-term breeding objective.

## Teaser

Cow longevity is an economically important trait for Australian beef breeders that requires further genetic improvement. Random regression models can enhance the genetic evaluations of longevity if longevity is considered a long-term breeding goal.

## Introduction

Cow longevity is a complex, economically important trait for beef cattle breeding programs. It directly relates to lifetime productivity and farm profitability by decreasing the ratio of rearing costs to production profit per animal. Improving longevity leads to improved animal welfare and more sustainable beef production systems. Improving cow longevity also impacts breeding programs by reducing the number of replacement heifers required, for a constant herd size, which provides more opportunities for voluntarily culling based on animal performance (Van Arendonk, 1985). Despite its great importance, early selection for longevity is complex because the true longevity of a cow is not known until the end of her life.

Different definitions of longevity have been used in livestock. In cattle, the most common measurements are lifetime and stayability traits (see review by Schuster et al., 2020). Lifetime measurements are continuous and have been traditionally defined as herd life which is the time from birth to culling of an animal. Another example of lifetime traits is the length of productive life which only comprises the period after first calving until culling (Nieuwhof et al., 1989). Stayability traits define a binary variable of survival (success or failure usually recoded as 1 and 0, respectively) between different time points (Jairath et al., 1998). These time points are often consecutive calvings or certain ages of animals. Longevity features that incorporate the productivity of animals during their lifetime (defined as functional longevity (FL)) are the preferred measures since production has a large impact on culling decisions especially in early life (Dürr et al., 1999).

Survival analysis using a proportional hazard model (PHM) is a popular method for the genetic evaluation of longevity traits (Ducrocq and Sölker, 1998). The PHM can accommodate the non-normal distribution of survival data, fit time-dependent environmental effects, and extrapolate the early survival data for the prediction of expected lifespan. In Australia, survival analysis using a Weibull PHM has been shown to provide an appropriate statistical framework for the analysis of the length of productive life in Angus cows, while the importance of a proper recording scheme needed for such analysis was highlighted (Meyer, 2009). If longevity is defined as binary traits then, in terms of statistical correctness, threshold models are optimal for the analysis of such binary response variables (Gianola and Foulley, 1983). However, linear models have been commonly employed in genetic evaluations of binary measures of longevity (Boettcher et al., 1999). Studies comparing the use of a linear versus a threshold model for the analysis of binary definitions of longevity have generally found little differences in genetic parameters and ranking of animals’ EBV between the 2 types of models (Sasaki et al., 2015; van Pelt et al., 2015 ). Linear models, therefore, seem to be suitable alternatives to threshold models as they are computationally less demanding (Sasaki et al., 2015) and more popular in routine genetic evaluations (Forabosco et al., 2009).

Veerkamp et al. (2001) showed the equivalence between a PHM and a random regression model (RRM) when applied to the binary representation of survival data (stayability). Following their work, linear RRM have been presented as an attractive method for the analysis of survival data (Jamrozik et al., 2013; Sasaki et al., 2015; van Pelt et al., 2015), as they can incorporate multiple measurements of longevity lifetime and fit multiple genetic effects parsimoniously so that longevity can be modeled as a different but genetically correlated trait across the entire lifespan of a cow. The use of multiple records for genetic evaluation of longevity guarantees the inclusion of all available information in the estimation of genetic parameters and improves the stability and accuracy of EBV at any point in the trajectory (Sánchez-Castro et al., 2019). In addition, a larger sample size allows for a more accurate correction of systematic environmental effects which implies that results are more relevant for practical applications as they provide more reliable estimates of genetic parameters (Heise et al., 2016).

The results from RRM were further compared to those from PHM where some studies found advantageous features of RRM and suggested them as the method of choice for the genetic evaluation of longevity traits. For example, Jamrozik et al. (2008) reported better predictive ability of RRM compared to PHM and multi-trait models when applied to the analysis of a simulated dataset of survival. Similarly, van Pelt et al. (2018) compared the EBV obtained from complete data and those from censored datasets for survival until 72 mo in dairy cattle and showed that the ranking of bulls based on breeding values estimated using an RRM were more stable than those obtained from a PHM, as more information was added in the analysis. RRM can combine information from other correlated traits in the genetic evaluation of longevity (Jamrozik et al., 2013) and provide a straightforward estimation of genetic correlations between longevity and other economically important traits. This is important as selection for longevity should consider its genetic correlations with other traits already under selection.

Longevity analyses are often impacted by censoring where there is no culling event recorded for animals that are still alive and hence a selected dataset which only includes the culled animals is used for the estimation of breeding values. This can unfavorably impact the accuracy of breeding values, especially for young bulls for which a high proportion of their daughters are still alive (Hou et al., 2009). The use of incomplete daughter information for estimation of breeding values might also deteriorate the rate of genetic progress as it can lead to lower rankings of younger bulls compared to older bulls and therefore the selection of later in breeding programs which tend to have lower genetic merits than the former (Oliveira et al., 2021). In addition, as true longevity is measured late in life, the generation interval is long and this hinders the genetic improvement of longevity. Genetic evaluation models that can incorporate information on live animals and use earlier measures of longevity can improve the prediction of genetic merits and facilitate the early selection of animals. RRMs are capable of using missing observations in the analysis for animals that are still alive (Schaeffer, 2003) and include any available information in the analysis without the need to have the same number of records for all animals.

The objective of this study was to apply RRM for the estimation of variance components and genetic parameters for cow longevity in Australian Angus cattle population. Two different measures of stayability (i.e., traditional and FL) across consecutive ages of 2 to 11 were examined in this study. Genetic correlations between different ages were estimated to inform the genetic relationships between longevity measures in early and late life. We also investigated the impact of censored data on estimated breeding values (EBV) of bulls with different proportions of active daughters.

## Materials and Methods

Animal Ethics approval was not required for this study since the information was obtained from data recorded under commercial conditions as part of routine management.

### Data

Culling, calving, and pedigree records of 1,438,363 cows were extracted from Angus Australia database. The following data cleaning protocols were used. Animals with missing date of birth, date of culling and those with multiple culling records were removed. Cows born before 2000 and those with an age at culling of less than 19 mo or greater than 20 yr were excluded. The progeny records with a missing date of birth, missing sire, and those from an embryo transfer were removed. A maximum of 2 calving events per yr was permitted for cows with multiple annual calving. For twin calves, only one calf was retained. Only calving records from cows with an age at first calving between 19 and 30 mo were kept. To minimize the impact of incorrect culling and calving dates, a maximum of 2 missing calvings was permitted between 2 reported calving events and between the last reported calving and culling records for each cow. This resulted in a total of 556,813 real calving events recorded for 176,361 cows with 25 unique culling reasons. Following the approach used by Oliveira et al. (2020), individual culling reasons supplied by breeders were categorized into 4 culling groups with known culling reasons including culling because of fertility (FERTIL), natural death (NDEATH), structural problems (STRUCT), and animal performance (PERFOR) and one group with all known and unknown culling reasons (ALLRES). The details of the different culling groups are shown in Table 1. The pedigree of animals with phenotypes was traced back to 4r generations and included a total of 373,475 animals.

**Table 1.** Number of animals culled in different groups

Culling group	Number of cows
Fertility (FERTIL)	36,472
Natural death (NDEATH)	24,867
Structural problems (STRUCT)	4,538
Performance (PERFOR)	3,847
All known and unknown culling reasons (ALLRES)	176,361
ALLRES plus Active (ALLACT)	223,139

### Phenotypes

**Figure 1.**
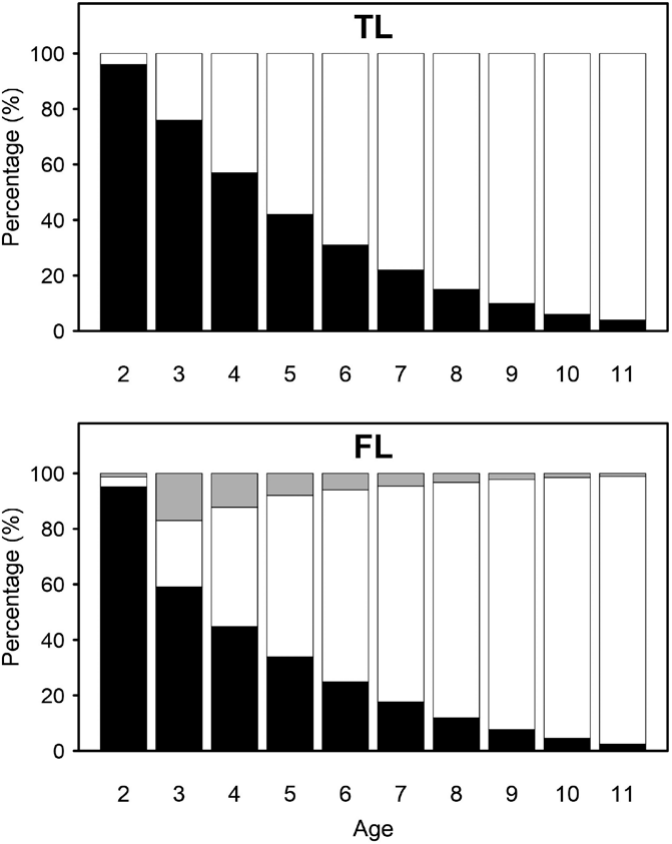
The percentage of different values (1: solid black; 0: solid white; and missing: solid gray) for traditional (top) and functional (bottom) longevity traits across ages 2 to 11 yr.

Longevity was defined as stayability to consecutive yearly ages following Oliveira et al. (2020). Animals which were present in the herd after first calving were coded as 1 at age *t* if the animal avoided culling from age *t-1* to age *t* and as 0 if the animal was culled before age *t*. This was regarded as the traditional longevity (TL) trait. To account for calving events during the life of a cow, an additional FL trait was defined by replacing the TL records at age *t* by missing observations, when there was no calving reported between age *t-1* and *t*. Initial investigation showed almost no variation in records after age 11 yr. Therefore, for each animal 10 binary records were assigned from age 2 to 11 yr. The proportions of different values for each longevity definition across ages 2 to 11 yr are shown in Figure 1.

### Statistical model

A single-trait RRM was fitted to estimate variance components for each culling group and longevity definition separately, using a Bayesian Gibbs sampler from gibbs3f90 software (Misztal et al., 2014):


$$\begin{array}{l} y=Xb+Hh+Za+Wp+e \end{array}$$ (1)

where **y** is the vector of phenotypes; **b** is the vector of estimates for overall mean, the fixed class effect of embryo transfer of cow birth and fixed regression coefficients for year-season of cow birth; **h**, **a,** and **p** are vectors of random regression coefficients for herd of calving within year-season of cow birth (herd-year-season), animal additive genetic and permanent environment deviations, respectively; **e** is the vector of random residuals and **X**, **H**, **Z,** and **W** are incidence matrices for the corresponding effects in the model. The general distribution of phenotypic records was assumed as $y|b,h,a,p,\Sigma_{h},\Sigma_{a},\Sigma_{p},\Sigma_{e}∼N\left(Xb+Hh+Za+Wp,I_{e}⊗\Sigma_{e}\right)$, where **Σ**_**h**_, **Σ**_**a**_, and **Σ**_**p**_ are the matrices of (co)variance components for random regression coefficients of **h**, **a,** and **p**, respectively, and **Σ**_**e**_ is the random residual (co)variance matrix which was assumed to be heterogeneous (i.e., ten classes, one each for each age). A uniform prior distribution was assumed for **b** as $b∼N(0,I_{b}⊗\Sigma_{b})$ and **h**, **a**, **p,** and **e** were assumed to be distributed as $h|\Sigma_{h}∼N(0,I_{h}⊗\Sigma_{h})$, $a|\Sigma_{a}∼N(0,A⊗\Sigma_{a})$, $p|\Sigma_{p}∼N(0,I_{p}⊗\Sigma_{p})$ and $e|\Sigma_{e}∼N(0,I_{e}\Sigma_{e})$, respectively, where **Σ**_**b**_ is a diagonal matrix with large values to represent non-informative prior knowledge, **I**_**b**_**, I**_**h**_, **I**_**p**_ and **I**_**e**_ are identity matrices and **A** is the numerator relationship matrix. The inverse Wishart distribution was adopted for **Σ**_**h**_, **Σ**_**a**_, and **Σ**_**p**_, whereas a scaled inverted chi-squared distribution was assumed for $\Sigma_{e}$.

Initial analyses were undertaken to determine the appropriate systematic effects and the optimal number of Legendre polynomials to use in the model (results not shown). It was decided to fit the same order of 4 Legendre polynomials for both fixed and all random regression terms in the final analyses. A total of 150,000 Markov Chain Monte Carlo (MCMC) chains with a burn-in of 50,000 and a thinning of 10 were implemented in this study. Initial runs with a higher number of chains (500,000) and larger burn-ins (250,000) did not impact the final posterior means, so the shorter chains were used for efficiency. The convergence of MCMC samples was checked by visual inspection of trace plots of samples, the effective sample size after burn-in and the Geweke criterion (Geweke, 1992), implemented in POSTGIBBSF90 from the BLUPF90 family of programs (Misztal et al., 2014).

### Variance components, genetic parameters, and breeding values

The (co)variance component matrices of **Σ**_**h**_, **Σ**_**a**_, and **Σ**_**p**_ were calculated as the posterior means of 10,000 thinned MCMC-generated samples after burn-in. The herd-year-season (**G**_**h**_), additive genetic (**G**_**a**_), and permanent environmental (**G**_**p**_) (co)variance matrices at different ages were calculated as:


$$\begin{array}{l} G_{h}=\phi \Sigma_{h}\phi^{\prime },\;G_{a}=\phi \Sigma_{a}\phi^{\prime },\;and\;G_{p}=\phi \Sigma_{p}\phi^{\prime } \end{array}$$ (2)

where **ϕ** is a 10 × 5 matrix of independent covariates for fourth-order Legendre orthogonal polynomials (0, 1, …, 4) at 10 different ages (Kirkpatrick et al., 1990). Heritabilities and the proportion of other variance components over the total phenotypic variance at each age were calculated for each longevity trait and each culling reason group using the diagonal elements of the **G**_**h**_, **G**_**a**_, **G**_**p,**_ and **Σ**_**e**_ (co)variance matrices. For example, the heritability at age *t* (*t* = 2, 3, …, 11) was obtained as:


$$\begin{array}{l} {\hat{h}}_{t}^{2}=\frac{{\hat{\;}\sigma \;}_{a_{t}}^{2}}{{\hat{\;}\sigma \;}_{a_{t}}^{2}+{\hat{\;}\sigma \;}_{p_{t}}^{2}+{\hat{\;}\sigma \;}_{e_{t}}^{2}} \end{array}$$ (3)

where ${\hat{\;}\sigma \;}_{a_{t}}^{2},{\hat{\;}\sigma \;}_{p_{t}}^{2}\;\;\;and\;{\hat{\;}\sigma \;}_{e_{t}}^{2}$ are the additive genetic, permanent environmental, and residual variances at age *t* which together comprise the total phenotypic variance at this age. The genetic correlations (${\hat{r}}_{g}$) between different ages were calculated using the genetic (co)variance matrix of **G**_**a**_:


$$\begin{array}{l} {\hat{r}}_{g_{t_{i}t_{j}}}=\frac{{\hat{\;}\sigma \;}_{a_{t_{i}t_{j}}}}{\sqrt{{\hat{\;}\sigma \;}_{a_{t_{i}}}^{2}{\hat{\;}\sigma \;}_{a_{t_{j}}}^{2}}} \end{array}$$ (4)

where ${\hat{\;}\sigma \;}_{a_{t_{i}t_{j}}}$ is the genetic covariance between age *t*_*i*_ and *t*_*j*_ obtained from the off-diagonal elements of **G**_**a**_ and ${\hat{\;}\sigma \;}_{a_{t_{i}}}^{2}and\;{\hat{\;}\sigma \;}_{a_{t_{j}}}^{2}$ are the genetic variances at these ages. The EBV across different ages for animal *n* were obtained as:


$$\begin{array}{l} EBV_{n}=\phi {\hat{u}}_{n} \end{array}$$ (5)

where ${\hat{u}}_{n}$ is the vector of random regression coefficient solutions estimated for animal *n* (Kirkpatrick et al., 1990).

### Impact of censoring on EBV

To investigate the impact of censored data on the estimation of breeding values, an additional dataset (ALLACT in Table 1) was created by adding to the ALLRES dataset, the active animals which were born after 2010 and were performing in the same contemporary groups (i.e., herd-year-season) as of those from ALLRES. The EBV for FL obtained from the analysis of ALLACT was compared to those from ALLRES and the Pearson correlations between the 2 sets of EBV were calculated for common sires with at least 10 daughters (*n* = 4,054). The correlations were further studied for sires with different proportions of active daughters in the analysis. In addition, the proportion of common sires between the 2 sets of analyses was calculated for the top 100 and 500 sires based on EBV at age 4 yr.

## Results

The number of animals from different culling groups are shown in Table 1. The largest number of animals (>60%) used in this study didn’t have a specific culling reason and were included in the group of all culling reasons together (ALLRES). Among the animals with a known culling reason, fertility was the primary reason for culling (~21%) and natural death was the second largest culling group (~14%). Only around 2% of animals were indicated culled because of voluntary reasons, i.e., performance. The ALLACT group included 46,778 (~20%) active animals in addition to the culled animals in ALLRES.

**Figure 2.**
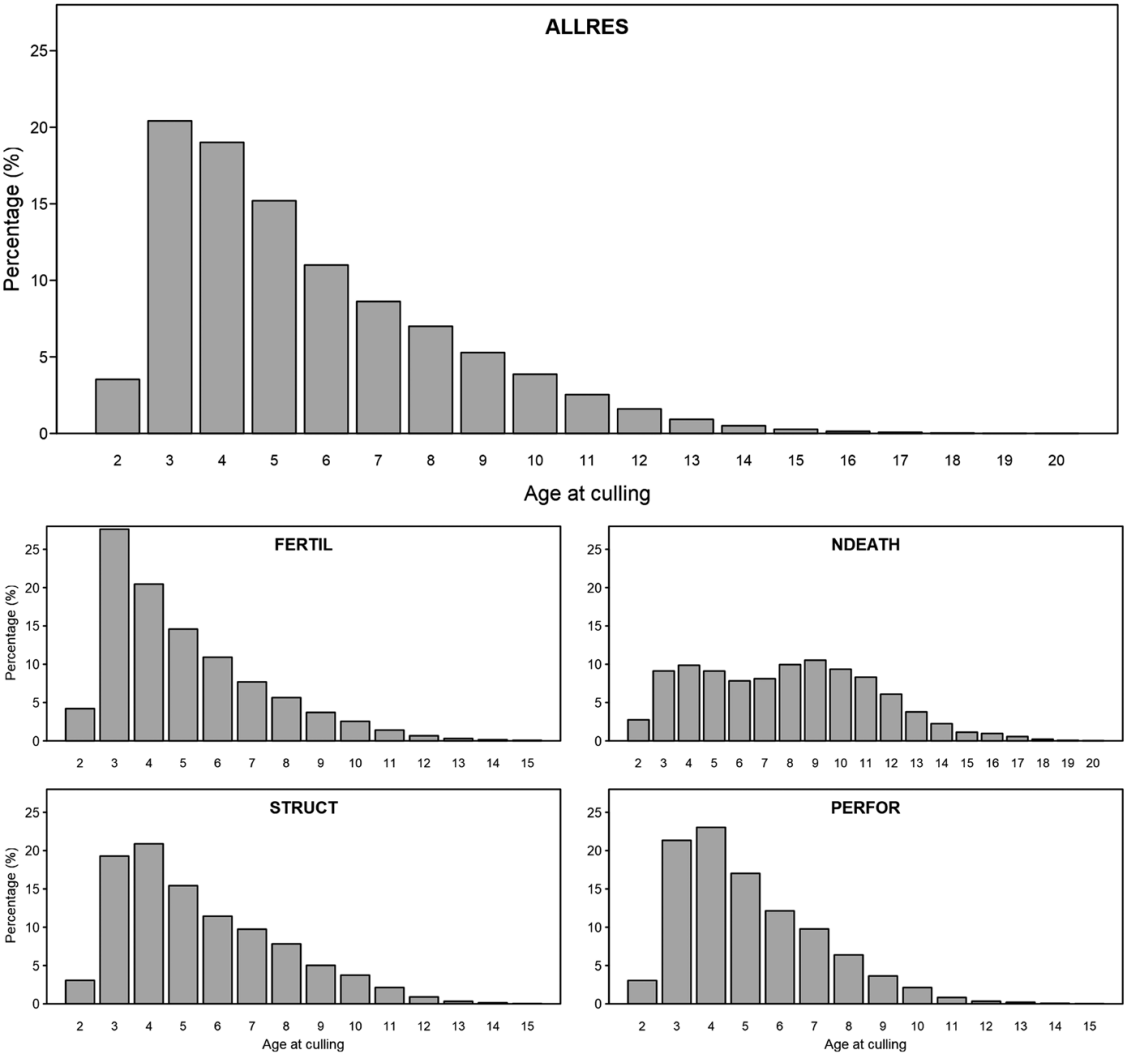
The percentage of animals culled at each age (yr) for different culling reason groups (ALLRES, all known and unknown culling reasons; FERTIL, fertility; NDEATH, natural death; STRUCT, structural problems; and PERFOR: performance).

The proportion of animals culled at each age for different culling reason groups are illustrated in Figure 2. Overall, more animals were culled at earlier ages after the age of 2 yr and the number of animals generally decreased towards the later ages (ALLRES). Culling because of fertility problems (FERTIL) tended to be carried out at an earlier age than culling because of other known reasons. On the other hand, animals survived longer in the natural death group (NDEATH). For STRUCT and PERFOR, more animals were culled at age 4 yr than any other age. Since the proportion of animals that were still alive after the age of 11 yr was very small for all culling reason groups with the exception of NDEATH (Figure 2), we decided to only include calvings between the ages of 2 and 11 yr in our analyses. This guaranteed the required contrast between the number of culled and alive animals at all ages and avoided estimates of variance at extreme ages where there was essentially no variation in the phenotype.

### Genetic parameters

#### Variance components

The estimated additive genetic, herd-year-season, and permanent environmental variances are illustrated in Figure S1-S3 for different culling reason groups and longevity definitions. The standard deviations of estimated variance components were small (≤0.01). For all culling groups and regardless of the longevity trait, the additive genetic variance was generally smaller compared to other estimated variance components across all ages. The permanent environmental variance explained the highest amount of variation across all ages and for all culling reason groups except STRUCT and PERFOR for which herd-year-season was the largest estimated variance component. For NDEATH, permanent environmental variance was larger in earlier ages while herd-year-season variance was estimated to be larger in later ages. Considering only the estimates of variance components across ages 4 to 6, the additive genetic variance was highest for FERTIL, STRUCT, and PERFOR at age 4, while it was highest at age 6 for NDEATH and at age 5 for ALLRES. Both the herd-year-season and permanent environmental variances were highest at age 4 for FERTIL, STRUCT, PERFOR, and ALLRES, but the highest estimates for NDEATH were obtained at age 6.

### Heritabilities

**Figure 3.**
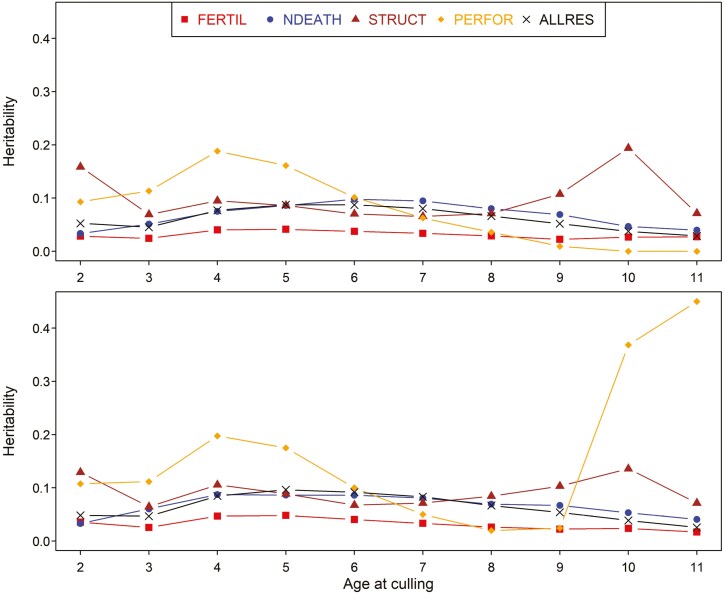
The estimated heritabilities for traditional (top) and functional (bottom) longevity traits and different groups of culling reasons (ALLRES, all known and unknown culling reasons; FERTIL, fertility; NDEATH, natural death; STRUCT, structural problems; and PERFOR, performance) across different ages (yr).

The estimated heritabilities for 5 groups of culling reasons and 2 longevity traits are shown in Figure 3. For both traditional and FL traits, the estimates of heritabilities were generally low and followed a similar pattern across different ages. Disregarding some large estimates at extreme ages (i.e., age 2 and 9 to 11 yr) for STRUCT and PERFOR, the peak heritabilities (±SD) of TL were found at age 5 yr for FERTIL (0.04 ± 0.00) and ALLRES (0.09 ± 0.00), at age 4 yr for PERFOR (0.19 ± 0.01) and STRUCT (0.10 ± 0.00) and at age 6 yr for NDEATH (0.10 ± 0.00). For FL, maximum heritabilities (± SD) were found at age 5 yr for FERTIL (0.05 ± 0.00) and ALLRES (0.10 ± 0.00) and at age 4 yr for PERFOR (0.20 ± 0.01), STRUCT (0.11 ± 0.00), and NDEATH (0.09 ± 0.00). Between all groups of culling reasons, the group with voluntarily culling reasons i.e., PERFOR achieved the highest heritability while FERTIL showed the lowest heritability. The heritabilities obtained from all groups of culling reasons (ALLRES) were more similar to those from NDEATH than any other known culling reason group.

### Genetic correlations

The estimated genetic correlations between different ages are given in Table 2 for the ALLRES group and in Tables S1-S4 for all other culling reason groups. As expected, for all groups of culling reasons and both longevity traits, genetic correlations were generally high between adjacent ages but decreased as the distance between ages increased. Genetic correlations were smaller for STRUCT and PERFOR and decreased rapidly as the distance between ages increased such that negative genetic correlations were found between ages that were furthest apart i.e., early and late ages. Conversely, genetic correlations remained high between different ages, even if they were distant, when animals died because of natural death (NDEATH). Genetic correlations between ages 3 to 5 yr and age 6 yr were moderate to high and ranged between 0.91 and 0.99 for ALLRES, between 0.81 and 0.98 for FERTIL, between 0.72 and 0.98 for NDEATH, between 0.57 and 0.94 for STRUCT and between 0.90 and 0.99 for PERFOR culling reason groups.

**Table 2.** Genetic correlations between different ages (yr) obtained for TL (upper diagonal) and FL (lower diagonal) traits from ALLRES dataset

Age (yr)	2	3	4	5	6	7	8	9	10	11
2		0.57	0.45	0.39	0.36	0.33	0.30	0.27	0.24	0.23
3	0.53		0.98	0.95	0.91	0.85	0.79	0.72	0.67	0.67
4	0.42	0.99		0.99	0.96	0.92	0.86	0.80	0.74	0.74
5	0.39	0.97	0.99		0.99	0.96	0.92	0.86	0.80	0.80
6	0.36	0.93	0.97	0.99		0.99	0.96	0.92	0.85	0.85
7	0.34	0.88	0.93	0.96	0.99		0.99	0.96	0.90	0.89
8	0.31	0.81	0.87	0.92	0.96	0.99		0.99	0.94	0.93
9	0.26	0.74	0.80	0.86	0.92	0.96	0.99		0.98	0.97
10	0.23	0.69	0.75	0.82	0.88	0.93	0.97	0.99		0.99
11	0.26	0.66	0.72	0.78	0.84	0.89	0.93	0.96	0.98	

TL, Traditional longevity; FL, Functional longevity; ALLRES, all known and unknown culling reasons.

### Impact of censoring on EBV

The correlation between FL EBV obtained from ALLRES and ALLACT datasets averaged across ages 2 to 11 yr was 0.97 for all animals. The correlation for sires with at least 10 culled daughters but with no active daughter was 0.92 (results are not shown). Table 3 shows the correlations between EBV of sires with active daughters who had at least 10 daughters in the analyses. As the minimum proportion of active daughters increased, the correlation between sire EBV from the 2 sets of analyses decreased. Correlations were generally higher in earlier ages and became lower in later ages. The average EBV at age 4 yr were always higher in ALLACT than those from ALLRES datasets and their difference increased as sires had more active daughters in the analysis. The proportion of common sires between ALLRES and ALLACT analyses is shown in Table 4. The proportion of common sires was low for both the top 100 (~2.5%) and top 500 (~12%) sires, but a higher proportion of common sires were found among the top 500 in both analyses. The ALLACT analysis resulted in a higher average EBV of top sires at age 4 yr when considering both top 100 and top 500 sires.

**Table 3.** The correlations between EBV of sires for FL obtained from ALLRES and ALLACT datasets with different proportions of active daughters

Minimum proportion of active daughters	Age at culling, yr										Average EBV at age 4 yr	
	2	3	4	5	6	7	8	9	10	11	ALLRES	ALLACT
0.1	0.85	0.76	0.76	0.74	0.73	0.71	0.68	0.66	0.65	0.64	0.18	0.20
0.3	0.81	0.71	0.70	0.70	0.67	0.65	0.62	0.60	0.58	0.58	0.18	0.22
0.5	0.77	0.68	0.68	0.67	0.66	0.64	0.62	0.60	0.59	0.59	0.19	0.23
0.7	0.68	0.60	0.60	0.60	0.59	0.57	0.55	0.53	0.52	0.52	0.19	0.25
0.9	0.65	0.58	0.58	0.57	0.55	0.53	0.51	0.50	0.49	0.50	0.19	0.26

EBV, Estimated breeding values; FL, Functional longevity; ALLRES, all known and unknown culling reasons; ALLACT, ALLRES plus active animals.

**Table 4.** The proportion of common sires between ALLRES and ALLACT based on EBV at age 4 yr

Number of top sires	Proportion of common sires	Average sire EBV ALLRES	Average sire EBV ALLACT
100	0.36	0.30	0.37
500	0.59	0.26	0.30

ALLRES, all known and unknown culling reasons; ALLACT, ALLRES plus active animals; EBV, estimated breeding value.

## Discussion

Longevity is an economically important trait in beef cattle production directly impacting farm profitability. Traits related to longevity are becoming more popular in routine genetic evaluations of beef cattle breeding programs (American Angus Association, 2023). Longevity measures that better suit the beef cattle production systems together with statistical methods capable of using information from multiple measurements across the entire life as well as from live animals can help with breeding for longer-lasting beef cattle. Here, 2 binary measures of cow longevity from age 2 to 11 yr were analyzed using a linear RRM to estimate variance components and genetic parameters of longevity in Australian Angus cattle. The impact of including censored records on EBV of sires with different proportions of active daughters was also investigated. Heritabilities were generally low and the genetic correlations between different ages indicated that longevity at early and late life are not genetically identical traits. Our results showed that censoring can unfavorably impact the breeding values of sires for longevity causing large re-rankings for selection purposes.

### Variance components and heritabilities

In this study, we estimated additive genetic, permanent environment, and herd-year-season variances for each group of culling reasons separately, as well as for the group of all culling reasons combined. The posterior means of variance components showed that the additive genetic variance was generally smaller than other variance components at all ages regardless of the culling group. This resulted in low estimates of heritabilities ranging between 0.02 and 0.19 for TL and between 0.02 and 0.20 for FL. Some large estimates of heritabilities at extreme ages were found for PERFOR and STRUCT which were the artifact of close-to-zero phenotypic variance in these ages and should therefore be disregarded. The PERFOR and STRUCT were the smallest datasets used in this study and in these groups, there were very small numbers of culled or alive animals at early and late ages, respectively, which contributed to a lack of variation in these ages. Unstable estimates of variance components at extreme points have been observed in other studies applying RRM with polynomial (co)variance functions in livestock (Pool and Meuwissen, 2000).

The large estimates of non-genetic variance components across all ages for both longevity traits and in all culling groups indicated that longevity is mainly controlled by environmental factors. The permanent environmental variance explained the highest amount of variation across all ages for most culling reason groups. This suggests that the environmental influences on longevity are largely individual-specific, i.e., environment impacts the longevity of each animal differently. For STRUCT and PERFOR, herd-year-season was the largest estimated variance component. This may imply that there is a large variation among herds in how they emphasize culling for structural and performance traits.

Our results of small additive genetic variances and low heritabilities and large estimates of environmental variances for cow longevity in the Australian Angus population are in general agreement with other findings in the literature. Longevity-related traits in cattle have been commonly found with low heritabilities and are mostly determined by environmental influences (see review by Sasaki, 2013 for dairy cattle). In beef cattle, low values for estimates of heritabilites for longevity-related traits are very common. Brzáková et al. (2019) used linear models to estimate heritabilities of 2 longevity traits in a mixed-breed beef population of Czech Republic and reported values ranging between 0.05 and 0.08. Varona et al. (2012) used a threshold model and found a heritability of 0.050 for survival in Prienaica beef cattle. Morales et al. (2017) found larger heritabilites of 0.14 for 2 measures of cow longevity in Retinta breed from a survival analysis using PHM.

Despite the consensus that longevity is a lowly heritable trait in cattle, Oliveira et al. (2020) reported some large heritabilities for longevity traits of North American Angus cattle. They estimated variance components for 3 binary measures of cow longevity between ages 2 to 15 yr in 6 different culling reason groups and reported substantially larger heritabilities than our estimated values for all common culling reason groups and for both traditional and FL traits. Their peak heritabilities were larger than 0.4 for structural problems, larger than 0.3 for natural death and larger than 0.1 for fertility, performance, and all culling reasons together. These authors also found that the additive genetic variance was larger than the other variance components for the group of structural problems. However, they did not provide a plausible explanation for their high values for estimates of additive genetic variances and heritabilities which differed from most findings in the literature.

In the current study, the lowest and highest heritabilities were obtained in the FERTIL and PERFOR datasets, respectively. Generally, fertility-related traits in beef cattle have low heritabilities, while performance traits tend to have higher heritabilities (Mu et al., 2016). This suggests that longevity based on different culling reasons may be linked to the heritable traits that influence culling decisions, with these traits contributing to the genetic factors affecting longevity. Consequently, longevity based on different culling reasons might serve as a proxy for the actual traits underlying culling decisions. However, it is important to note that confirming this hypothesis would require estimating genetic correlations with the actual traits.

The genetic evaluation of longevity considering different culling reasons is expected to provide useful tools for breeders to select animals that are resilient against specific culling causes. However, one of the key challenges for such analyses is that when making culling decisions breeders often have more than one reason to cull an animal. Predefined disposal codes provided by breeding organizations may not include all reasons and this can result in the high proportion of unknown culling reasons, the highest group of culling reasons in our study, which cannot be used in obtaining longevity EBV specific to culling reasons. Culling decisions are made subjectively by breeders and different breeders may have different opinions to cull or keep the same animal. This can result in animals with the same genetic background for longevity being presented in different groups for culling reasons. In addition, the relative importance of different culling reasons can change over time and consequently, this influences the genetic background of culling (Heise et al., 2016). The direct genetic improvement of the underlying culling reasons which usually have higher heritabilities may improve longevity based on different groups of culling reasons accordingly. The genetic improvement of longevity based on all culling reasons together is concerned about the improvement of overall survival which is not achieved by the genetic improvement of the underlying culling reasons individually. Given these, genetic evaluations of longevity considering different culling reasons might not have real practical applications in breeding programs and combining all culling reasons together seems to be a better approach.

### Genetic correlations

As expected, genetic correlations estimated by RRM were high between consecutive ages and decreased as the distance between ages increased such that very low genetic correlations were obtained between some distant ages. The high genetic correlation between ages of close proximity has been attributed to the contribution of similar genetic factors impacting longevity at these ages which change between ages further apart (Sasaki et al., 2015). An exception in our study was age 2 yr for which low to moderate genetic correlations with earlier ages and unstable genetic correlations with late ages were found in some culling reason groups. Oliveira et al. (2020) observed similar patterns of genetic correlations between age 2 yr and older ages and suggested that the EBV obtained at age 2 yr are not suitable to improve longevity at older ages.

For both longevity traits, the patterns of genetic correlations between different ages and in all culling reason groups indicated that longevity evaluated at early and late life are not genetically the same traits. For example, considering ages 3 to 11 yr in ALLRES group, the genetic correlations quickly dropped below 0.9 for ages that were 4 or more yr apart and became as low as around 0.7 for some distant ages. These results are supported by previous studies who found low genetic correlations between longevity measures at distant time intervals. Jamrozik et al. (2013) reported a genetic correlation of 0.61 between stayability at age 2 and 8 yr in Canadian Simmental cattle. van Pelt et al. (2015) found that the genetic correlations from a linear RRM in Dutch dairy cattle were below 0.9 when there was a gap of 2 or more yr between survival measured after first calving. They also estimated genetic correlations of around 0.4 between survival measurements that were 5 yr apart. Heise et al. (2016) found a genetic correlations of 0.3 between survival traits of dairy cows at early first and late third lactations. Given the large impact of environment on longevity, these results indicate that environmental influences on longevity are not stable and change over time which in turn can change the genetic background of longevity during the lifespan of a cow causing longevity being a different trait in early and late life.

Survival to age 6 yr has been traditionally targeted in genetic evaluations of longevity in beef cattle as it has been identified as the break-even point when a cow pays for her replacement costs (Brigham et al., 2009). However, it has been criticized that the delayed recording of cow phenotypes until they reach the defined benchmark of 6 yr results in low accuracy of EBV in early life, especially for sires, and the absence of accurate predictions of longevity for selection of replacements when needed (Brigham et al., 2009; Jamrozik et al., 2013). Therefore, earlier measures of longevity have been suggested as indicator traits for the genetic prediction of longevity in late life. In this study, we used RRM to evaluate the genetic correlations between longevity measures at an extended trajectory from age 2 to 11 yr. For the group of all culling reasons together, the genetic correlations between earlier ages of 3 to 5 yr and age 6 yr were higher than 0.9 for both TL and FL, indicating that the majority of cows that are alive and/or calving at earlier ages will be still in the herd and/or in production when they become 6 yr old. This suggests that the genetic improvement of longevity in Australian Angus cattle can benefit from selection at an earlier age by shortening the generation interval and obtaining a correlated response to selection at later ages. The optimal age for selection should be chosen by considering a high heritability to maximize the genetic gain for increased longevity.

### Impact of censoring on genetic evaluation of longevity

One of the main advantages of applying RRM in genetic evaluations of longevity is the ability to include censored data. Longevity analyses often suffer from censoring, that is at any point in time there is a substantial proportion of cows which are still alive and their records are usually excluded from the analysis. Methods to account for censored data have focused on the prediction of expected lifespan by extending records for cows that are still alive (VanRaden and Klaaskate, 1993; Brotherstone et al., 1997). With RRM, information from living cows can be treated as missing observations for the prediction of longevity EBV at any point in the trajectory without the need to have the same number of records for all animals.

The EBV of sires based on their daughters’ longevity is an important criterion for sire selection. With censoring, the records from living cows are excluded from the estimation of breeding values, leading to biased EBVs for young sires with only culled daughters in the analysis (Veerkamp et al., 2001). However, with the inclusion of censored records, the EBVs of young sires are expected to become unbiased, as all available data from both culled and living cows are used. Furthermore, under an RRM framework, breeding value estimation leverages genetic correlations between different ages, which are informed by complete daughter records, particularly from proven bulls, ensuring accurate and unbiased estimations of longevity breeding values across all ages.

In this study, we investigated the impact of censored data on EBV of sires with different proportions of active daughters by comparing our results between ALLRES and ALLACT datasets. We found that with the inclusion of censored data, the EBV from ALLACT are lowly correlated with those from ALLRES. The correlations between 2 sets of EBV were especially low at later ages and for sires with larger proportions of active daughters. The breeding values of sires with active daughters were underestimated when the censored data was excluded from the prediction of EBV. This implies that they will have lower rankings when incomplete daughter information is used in obtaining their EBV. The top 100 and 500 sires from ALLACT dataset had higher average EBV than those from ALLRES, with only 36% and 59% of top 100 and 500 sires were in common between the 2 datasets, respectively. This suggests that the genetic gain for longevity will be hindered if censored records are ignored, as sires with lower breeding values will be selected for breeding. Garcia et al. (2017) compared the predictive abilities of models with and without censored records in Nelore cattle and observed that the model excluding censored records of longevity from the analysis showed a lower prediction ability, lower accuracy of sire EBV and large re-ranking of sires compared to the models with censored data.

The use of genomic information may reduce the need for the inclusion of censored data (Oliveira et al., 2021). Genomic information can improve the accuracy of EBV for young sires and consequently reduce generation interval leading to an increased response to selection. This is especially beneficial for longevity-related traits as traits with low heritability benefit the most from genomic selection (Meuwissen et al., 2001). Leite et al. (2021) investigated the benefit of including genomic information on the prediction accuracy of survival traits in pigs and found that the prediction accuracy of EBV were higher through genomic prediction compared to a traditional pedigree-based evaluation.

## Conclusions

The genetic improvement of longevity in Australian Angus cattle is still possible if longevity is considered as a long-term breeding objective, even with the low heritabilities obtained in this study. The additive genetic component has a sizeable contribution to the variability of longevity which indicates that the genetic progress can be achieved as it is permanent and accumulative. The inclusion of censored records is expected to further increase the genetic progress for longevity as it leads to higher average breeding values of top sires. The patterns of genetic correlations between different ages indicated that longevity in early and late life are not genetically identical traits. Combining all culling reasons to obtain a single EBV for longevity can simplify implementation for breeders and provide practical benefits in breeding programs. The genetic improvement of longevity should consider its genetic correlations with other economically important traits and the inclusion of genomic information to maximize genetic gain for an increased longevity.

## Supplementary Material

skaf035_suppl_Supplementary_Figure_S1

skaf035_suppl_Supplementary_Figure_S2

skaf035_suppl_Supplementary_Figure_S3

skaf035_suppl_Supplementary_Material

## Abbreviations

ALLACT: ALLRES plus active animals
ALLRES: all known and unknown culling reasons
EBV: estimated breeding value
FERTIL: fertility
FL: functional longevity
MCMC: Markov Chain Monte Carlo
NDEATH: natural death
PERFOR: performance
PHM: proportional hazard model
RRM: random regression model
STRUCT: structural problems
TL: traditional longevity
