# Supplementary figures and images for: Genetic evaluation of longevity in Australian Angus cattle using random regression models
Source: J Anim Sci. 2025 Feb 8;103:skaf035. doi: 10.1093/jas/skaf035 (PMC11914885; doi:10.1093/jas/skaf035)

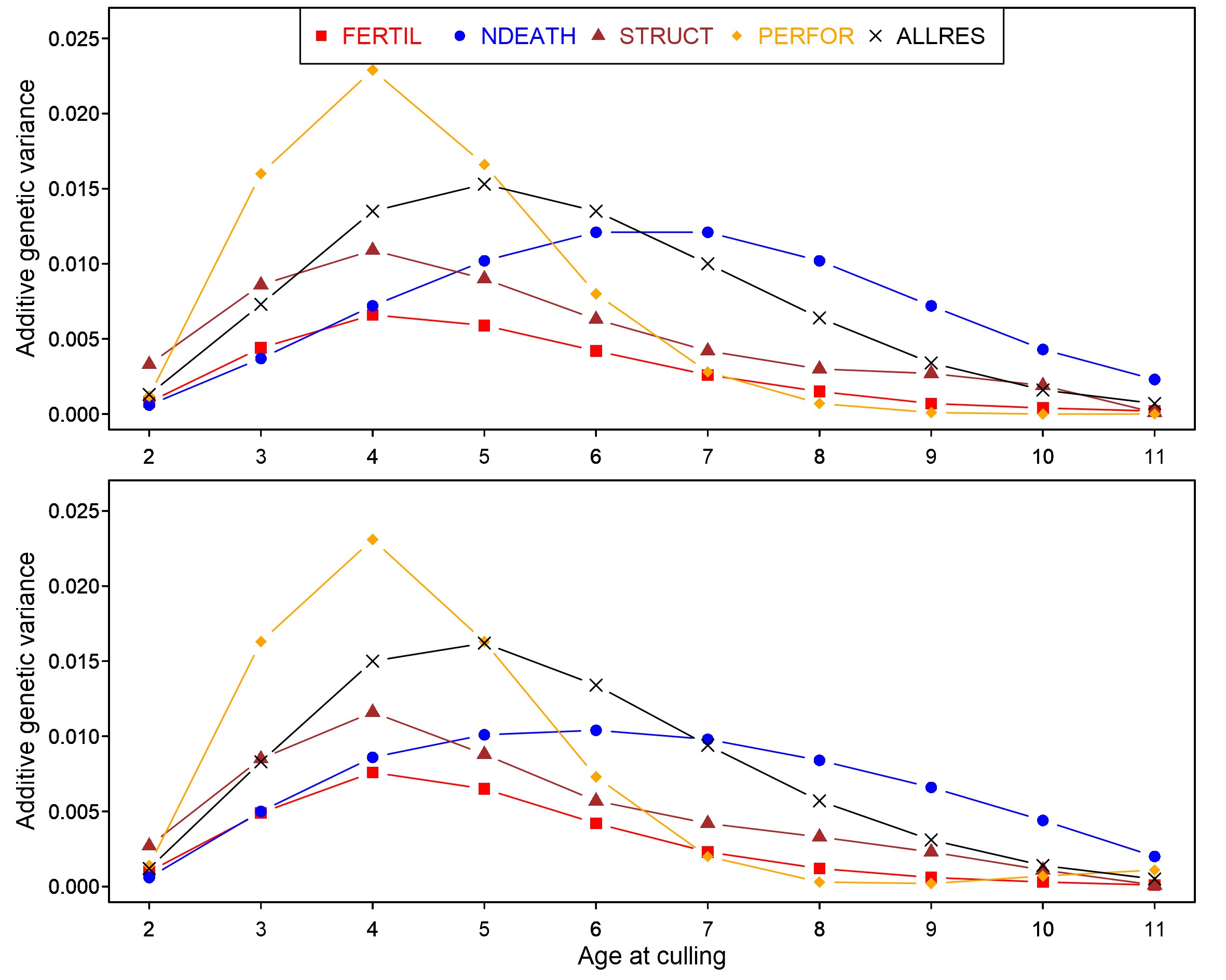

Supplement: skaf035_suppl_Supplementary_Figure_S1 [file skaf035_suppl_supplementary_figure_s1.jpeg]

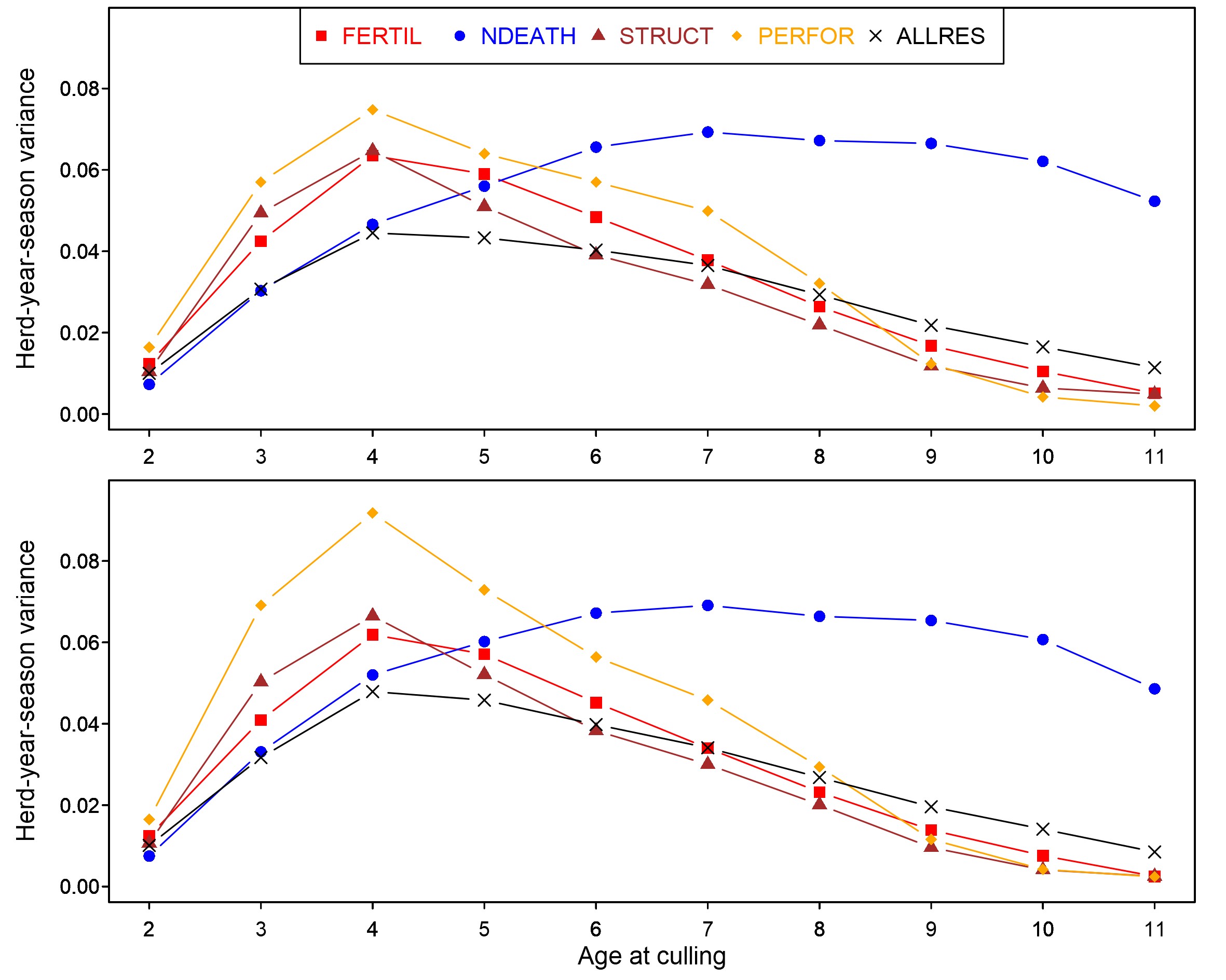

Supplement: skaf035_suppl_Supplementary_Figure_S2 [file skaf035_suppl_supplementary_figure_s2.jpeg]

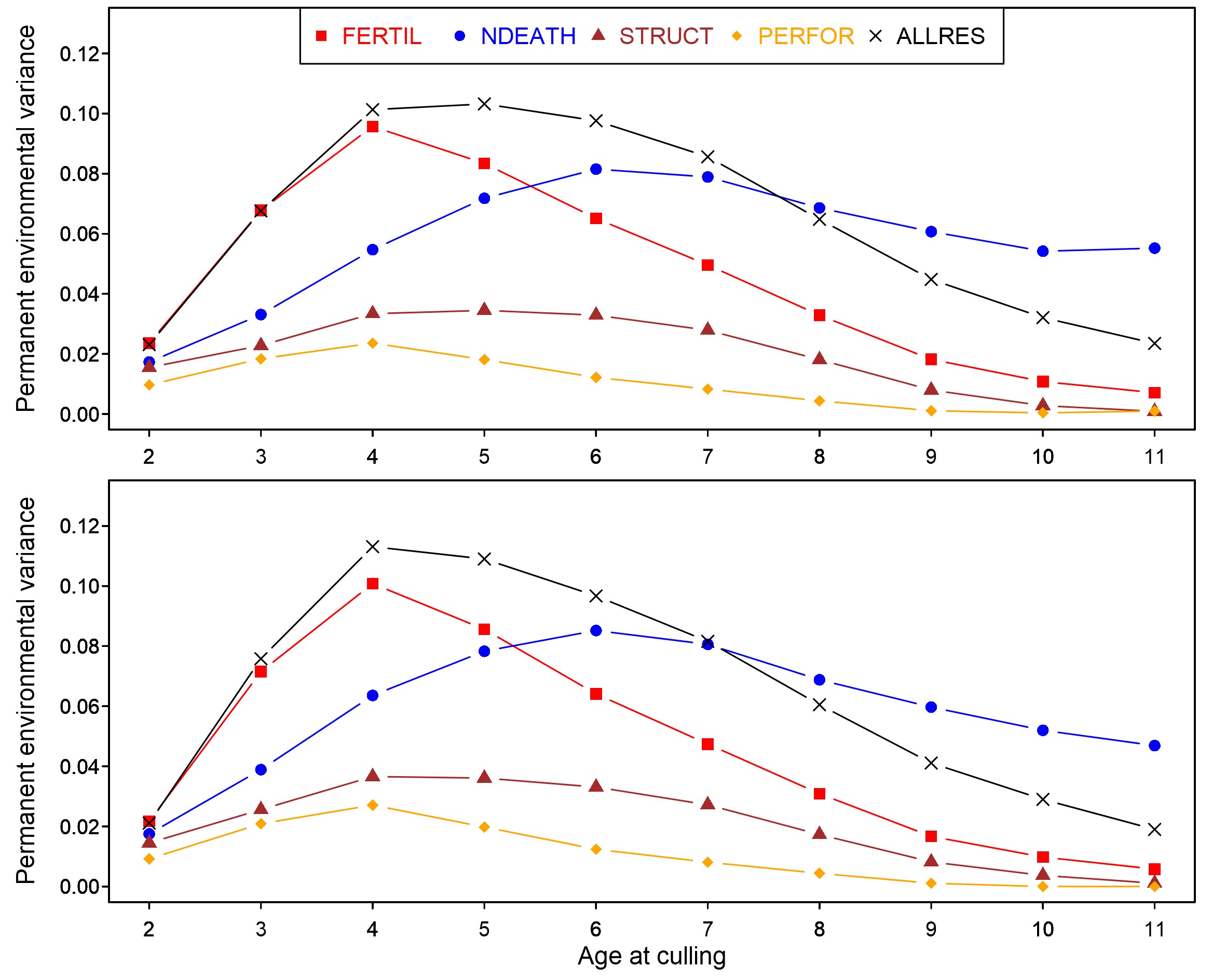

Supplement: skaf035_suppl_Supplementary_Figure_S3 [file skaf035_suppl_supplementary_figure_s3.jpeg]
