# Supplementary material for: Genetic evaluation of longevity in Australian Angus cattle using random regression models
Source: J Anim Sci. 2025 Feb 8;103:skaf035. doi: 10.1093/jas/skaf035 (PMC11914885; doi:10.1093/jas/skaf035)
Supplement: skaf035_suppl_Supplementary_Material [file skaf035_suppl_supplementary_material.docx]

**Genetic Evaluation of Longevity in Australian Angus cattle Using Random Regression Models**

Hassan Aliloo,^†1^ Julius H. J. van der Werf^†^ and Samuel A. Clark^†^

^†^School of Environmental and Rural Science, University of New England, Armidale, NSW, Australia

^1^Corresponding author. Email: haliloo@une.edu.au

**Supplemental Materials**

**Supplemental figure captions**

Figure S1. The estimated additive genetic variance for traditional (top) and functional (bottom) longevity traits and different groups of culling reasons (ALLRES: all known and unknown culling reasons; FERTIL: fertility; NDEATH: natural death; STRUCT: structural problems; and PERFOR: performance) across different ages (yr).

Figure S2. The estimated herd-year-season variance for traditional (top) and functional (bottom) longevity traits and different groups of culling reasons (ALLRES: all known and unknown culling reasons; FERTIL: fertility; NDEATH: natural death; STRUCT: structural problems; and PERFOR: performance) across different ages (yr).

Figure S3. The estimated permanent environmental variance for traditional (top) and functional (bottom) longevity traits and different groups of culling reasons (ALLRES: all known and unknown culling reasons; FERTIL: fertility; NDEATH: natural death; STRUCT: structural problems; and PERFOR: performance) across different ages (yr).

Table S1. Genetic correlations between different ages (yr) obtained for TL^1^ (upper diagonal) and FL^2^ (lower diagonal) traits from FERTIL^3^ dataset.

| Age (yr) | 2 | 3 | 4 | 5 | 6 | 7 | 8 | 9 | 10 | 11 |
| --- | --- | --- | --- | --- | --- | --- | --- | --- | --- | --- |
| 2 |  | 0.55 | 0.40 | 0.30 | 0.19 | 0.10 | 0.03 | 0.05 | 0.13 | 0.12 |
| 3 | 0.38 |  | 0.98 | 0.92 | 0.81 | 0.66 | 0.54 | 0.50 | 0.53 | 0.45 |
| 4 | 0.24 | 0.98 |  | 0.98 | 0.91 | 0.79 | 0.68 | 0.61 | 0.57 | 0.51 |
| 5 | 0.18 | 0.95 | 0.99 |  | 0.97 | 0.90 | 0.80 | 0.72 | 0.61 | 0.57 |
| 6 | 0.14 | 0.87 | 0.93 | 0.98 |  | 0.97 | 0.91 | 0.81 | 0.64 | 0.63 |
| 7 | 0.10 | 0.74 | 0.82 | 0.90 | 0.97 |  | 0.98 | 0.88 | 0.68 | 0.69 |
| 8 | 0.06 | 0.58 | 0.68 | 0.78 | 0.88 | 0.97 |  | 0.95 | 0.76 | 0.78 |
| 9 | 0.04 | 0.50 | 0.59 | 0.68 | 0.79 | 0.89 | 0.97 |  | 0.92 | 0.91 |
| 10 | 0.06 | 0.54 | 0.60 | 0.66 | 0.72 | 0.79 | 0.86 | 0.95 |  | 0.95 |
| 11 | 0.22 | 0.57 | 0.61 | 0.67 | 0.74 | 0.81 | 0.86 | 0.91 | 0.93 |  |

^1^TL: Traditional longevity.

^2^FL: Functional longevity.

^3^FERTIL: fertility.

Table S2. Genetic correlations between different ages (yr) obtained for TL^1^ (upper diagonal) and FL^2^ (lower diagonal) traits from NDEATH^3^ dataset.

| Age (yr) | 2 | 3 | 4 | 5 | 6 | 7 | 8 | 9 | 10 | 11 |
| --- | --- | --- | --- | --- | --- | --- | --- | --- | --- | --- |
| 2 |  | 0.76 | 0.64 | 0.54 | 0.47 | 0.42 | 0.42 | 0.46 | 0.54 | 0.59 |
| 3 | 0.71 |  | 0.96 | 0.85 | 0.73 | 0.63 | 0.56 | 0.53 | 0.53 | 0.58 |
| 4 | 0.59 | 0.97 |  | 0.97 | 0.89 | 0.81 | 0.74 | 0.67 | 0.62 | 0.64 |
| 5 | 0.48 | 0.87 | 0.96 |  | 0.98 | 0.93 | 0.87 | 0.79 | 0.71 | 0.69 |
| 6 | 0.38 | 0.72 | 0.87 | 0.97 |  | 0.99 | 0.95 | 0.87 | 0.78 | 0.72 |
| 7 | 0.31 | 0.59 | 0.75 | 0.89 | 0.98 |  | 0.99 | 0.93 | 0.84 | 0.75 |
| 8 | 0.29 | 0.49 | 0.65 | 0.80 | 0.91 | 0.98 |  | 0.98 | 0.91 | 0.79 |
| 9 | 0.33 | 0.45 | 0.58 | 0.72 | 0.83 | 0.91 | 0.98 |  | 0.97 | 0.84 |
| 10 | 0.41 | 0.49 | 0.57 | 0.67 | 0.76 | 0.84 | 0.92 | 0.98 |  | 0.90 |
| 11 | 0.52 | 0.64 | 0.73 | 0.80 | 0.85 | 0.88 | 0.91 | 0.92 | 0.94 |  |

^1^TL: Traditional longevity.

^2^FL: Functional longevity.

^3^NDEATH: natural death.

Table S3. Genetic correlations between different ages (yr) obtained for TL^1^ (upper diagonal) and FL^2^ (lower diagonal) traits from STRUCT^3^ dataset.

| Age (yr) | 2 | 3 | 4 | 5 | 6 | 7 | 8 | 9 | 10 | 11 |
| --- | --- | --- | --- | --- | --- | --- | --- | --- | --- | --- |
| 2 |  | 0.59 | 0.31 | 0.06 | -0.15 | -0.25 | -0.14 | 0.15 | 0.39 | 0.15 |
| 3 | 0.25 |  | 0.94 | 0.79 | 0.57 | 0.30 | 0.10 | 0.07 | 0.14 | 0.13 |
| 4 | 0.04 | 0.96 |  | 0.95 | 0.80 | 0.54 | 0.26 | 0.06 | 0.02 | 0.07 |
| 5 | -0.04 | 0.85 | 0.95 |  | 0.94 | 0.75 | 0.44 | 0.12 | -0.04 | 0.00 |
| 6 | -0.02 | 0.60 | 0.75 | 0.91 |  | 0.92 | 0.66 | 0.28 | 0.01 | -0.06 |
| 7 | 0.11 | 0.25 | 0.40 | 0.63 | 0.89 |  | 0.88 | 0.55 | 0.23 | -0.08 |
| 8 | 0.34 | 0.03 | 0.11 | 0.33 | 0.65 | 0.91 |  | 0.87 | 0.61 | -0.01 |
| 9 | 0.60 | 0.03 | 0.00 | 0.13 | 0.40 | 0.70 | 0.91 |  | 0.92 | 0.13 |
| 10 | 0.82 | 0.23 | 0.09 | 0.08 | 0.18 | 0.38 | 0.64 | 0.89 |  | 0.29 |
| 11 | 0.46 | 0.36 | 0.26 | 0.17 | 0.03 | -0.14 | -0.25 | -0.25 | -0.09 |  |

^1^TL: Traditional longevity.

^2^FL: Functional longevity.

^3^STRUCT: structural problems.

Table S4. Genetic correlations between different ages (yr) obtained for TL^1^ (upper diagonal) and FL^2^ (lower diagonal) traits from PERFOR^3^ dataset.

| Age (yr) | 2 | 3 | 4 | 5 | 6 | 7 | 8 | 9 | 10 | 11 |
| --- | --- | --- | --- | --- | --- | --- | --- | --- | --- | --- |
| 2 |  | 0.43 | 0.35 | 0.31 | 0.28 | 0.21 | 0.08 | -0.13 | -0.28 | -0.66 |
| 3 | 0.43 |  | 0.99 | 0.97 | 0.90 | 0.73 | 0.37 | -0.07 | 0.04 | -0.38 |
| 4 | 0.35 | 0.99 |  | 0.99 | 0.94 | 0.79 | 0.46 | 0.02 | 0.01 | -0.32 |
| 5 | 0.31 | 0.98 | 0.99 |  | 0.98 | 0.87 | 0.57 | 0.13 | -0.06 | -0.28 |
| 6 | 0.28 | 0.94 | 0.97 | 0.99 |  | 0.95 | 0.72 | 0.31 | -0.17 | -0.24 |
| 7 | 0.19 | 0.84 | 0.88 | 0.92 | 0.97 |  | 0.90 | 0.56 | -0.31 | -0.17 |
| 8 | -0.14 | 0.50 | 0.57 | 0.64 | 0.73 | 0.86 |  | 0.84 | -0.39 | -0.08 |
| 9 | -0.53 | -0.12 | -0.09 | -0.09 | -0.10 | -0.05 | 0.33 |  | -0.10 | -0.04 |
| 10 | -0.36 | -0.01 | -0.02 | -0.06 | -0.13 | -0.18 | 0.05 | 0.91 |  | -0.09 |
| 11 | -0.30 | -0.03 | -0.03 | -0.07 | -0.11 | -0.14 | 0.13 | 0.93 | 0.98 |  |

^1^TL: Traditional longevity.

^2^FL: Functional longevity.

^3^PERFOR: performance.


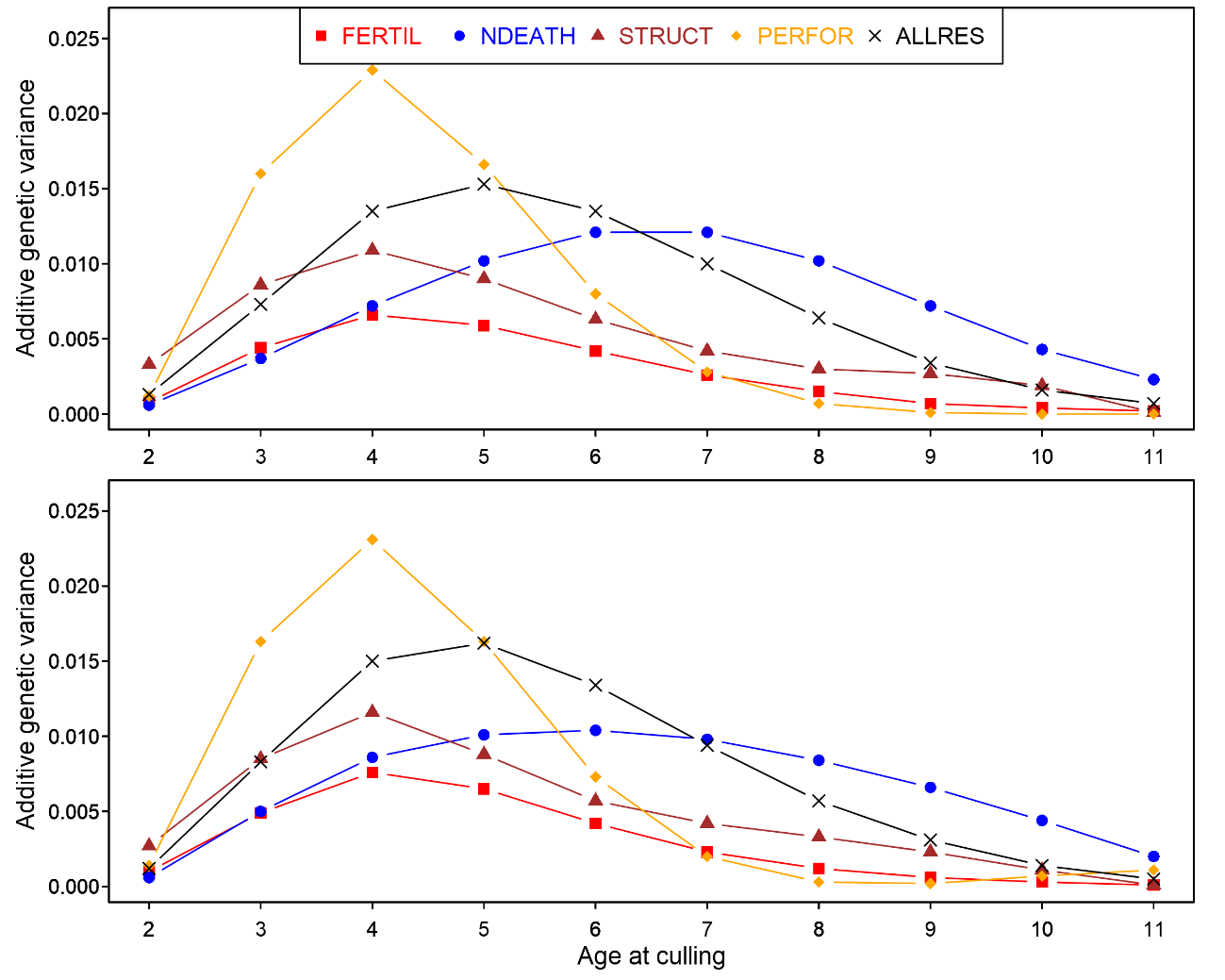


Figure S1. The estimated additive genetic variance for traditional (top) and functional (bottom) longevity traits and different groups of culling reasons (ALLRES: all known and unknown culling reasons; FERTIL: fertility; NDEATH: natural death; STRUCT: structural problems; and PERFOR: performance) across different ages (yr).


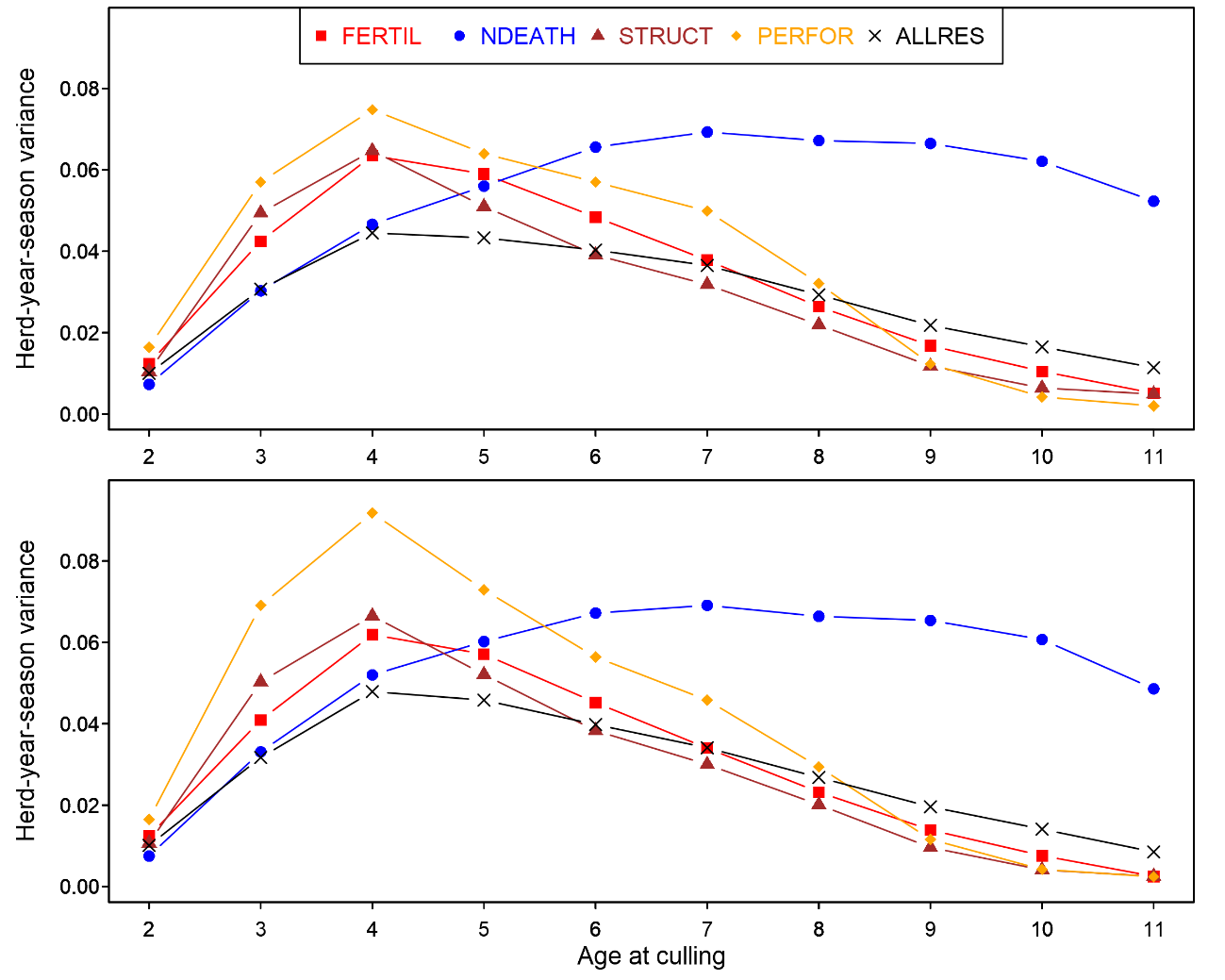


Figure S2. The estimated herd-year-season variance for traditional (top) and functional (bottom) longevity traits and different groups of culling reasons (ALLRES: all known and unknown culling reasons; FERTIL: fertility; NDEATH: natural death; STRUCT: structural problems; and PERFOR: performance) across different ages (yr).


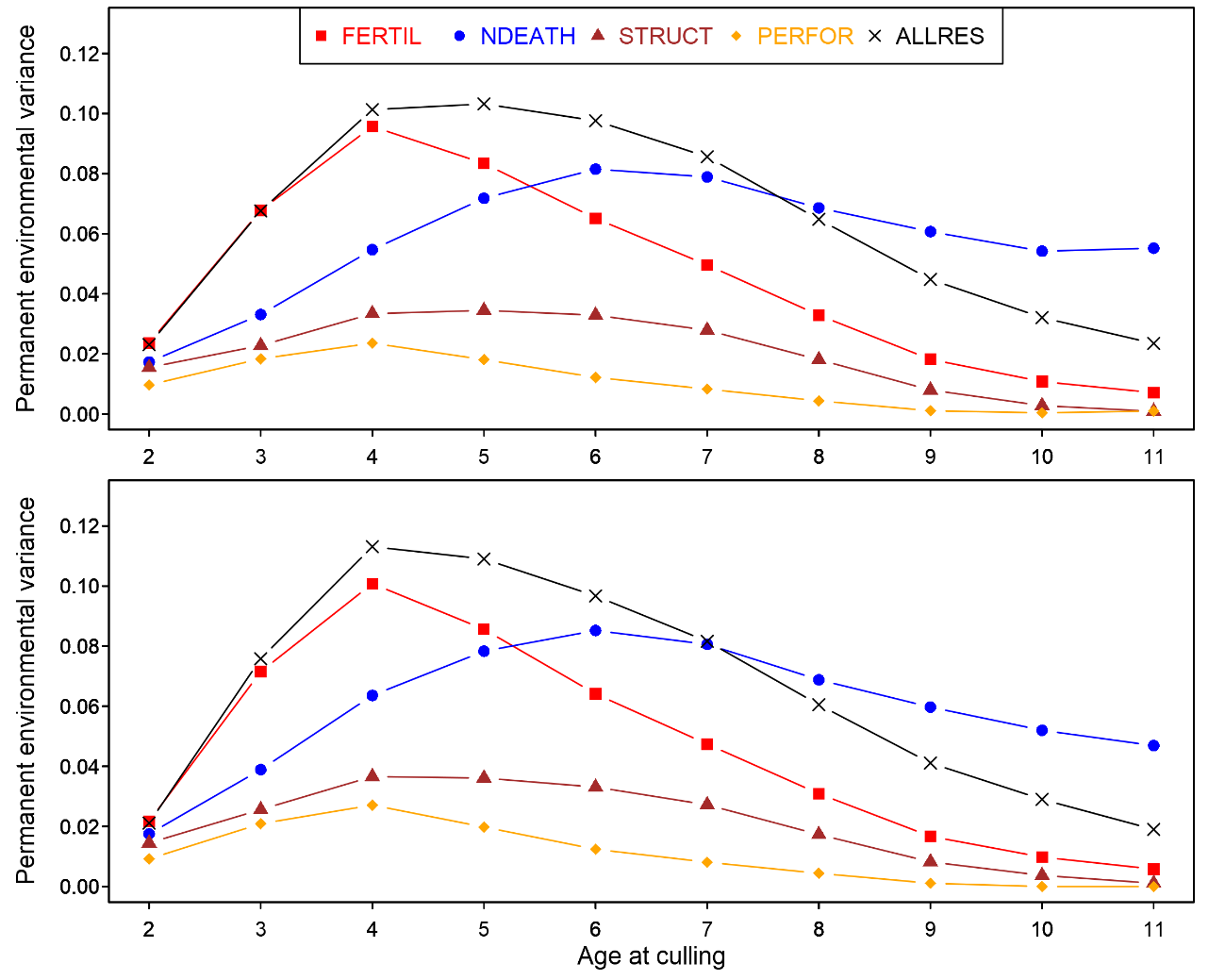


Figure S3. The estimated permanent environmental variance for traditional (top) and functional (bottom) longevity traits and different groups of culling reasons (ALLRES: all known and unknown culling reasons; FERTIL: fertility; NDEATH: natural death; STRUCT: structural problems; and PERFOR: performance) across different ages (yr).
